# Editorial: Computational models of affordance for robotics

**DOI:** 10.3389/fnbot.2022.1045355

**Published:** 2022-10-06

**Authors:** Erwan Renaudo, Philipp Zech, Raja Chatila, Mehdi Khamassi

**Affiliations:** ^1^Intelligent and Interactive Systems, Department of Computer Science, University of Innsbruck, Innsbruck, Austria; ^2^Quality Engineering, Department of Computer Science, University of Innsbruck, Innsbruck, Austria; ^3^Institute of Intelligent Systems and Robotics, Sorbonne Université, CNRS, Paris, France

**Keywords:** affordance learning, symbol emergence in robotics, babbling and exploration, sensorimotor contingencies, self-organization

Started and developed by Gibson, the affordance theory offered an alternative view to the fields of psychology and philosophy on the question of perception (Gibson, 1979). Gibson considered that we do not perceive our environment as a collection of objects described by their physical properties, but rather through the affordances it offers, i.e., the action possibilities of an agent to interact with its environment. For instance, a coffee mug affords humans the possibility to pour water into it, and then seize it by the handle and bring it to the mouth to drink. In contrast, the same mug affords rodents the possibility to hide behind it, or to climb on it to drink. Finally, the same mug affords insects the possibility to land on it. Thus the same object offers different affordances to different agents depending on their body properties, their sensorimotor abilities, and their cultural knowledge and habits.

The concept of affordance was subsequently refined by Gibson himself, as well as other researchers, in order to provide a common definition (Turvey, 1992; Greeno, 1994; Sanders, 1997; Steedman, 2002; Wells, 2002; Chemero, 2003; Michaels, 2003; Stoffregen, 2003). Such a definition had to cope with questions later raised within the community, such as whether affordances are attached to the environment only (Turvey, 1992) or to the agent-environment system (Steedman, 2002; Chemero, 2003; Stoffregen, 2003).

In parallel, the affordance theory fueled research in robot perception, which in return provided psychology with insights from some of the problems to which roboticists have been confronted. As pointed by Horton et al. (2012), the ecological view of perception had already influenced robotics, giving birth to the Reactive control paradigm which popularized the works of Brooks (1986) and Arkin (1990). Nevertheless, affordances were not explicitly mentioned in this paradigm. Later on, the formalization proposed by Sahin et al. (2007) targeted the explicit use of the concept of affordance for robot control. Sahin and colleagues reviewed the evolution of the concept, and defined affordances from *an agent's perspective* as acquired relationships between two equivalence classes: an effect equivalence class and an (environment entity, agent behavior) equivalence class. These relationships model “generally true knowledge” (but exceptions are possible) that allow the agent to predict the effects of its actions in novel situations. Affordances may thus play a pivotal role in generalization.

Later on, at the interface between computational neuroscience and robotics, researchers have emphasized the possibilities in terms of affordances offered by the mirror system in the primate brain (Thill et al., 2013). This neural system, organized around the premotor cortex, includes so-called *mirror neurons*, i.e., neurons which respond when a specific action is performed (e.g., grasping an object), independently from the agent that performs it (i.e., the same neuron responds both when the animal grasps an object and when it observes another agent performing the same action), and independently from the precise motor plan used to execute this action (i.e., the same neuron responds when the animal closes its hand to grasp the object, when it uses a tool such as pinch to grasp the object, and even when it uses an inverted pinch which tightens around the object when the animal opens its hand) (Rizzolatti et al., 2001). The mirror system thus appears to contribute to abstract representations of actions at a high level of a hierarchical cognitive architecture, in terms of the goal it offers (affords) to the agent (in the example, grasping an object) (Thill et al., 2013). Such representations would moreover be critical for the understanding of other agents' behavior, and could thus contribute to imitation and other social skills.

In our view, using affordances in robotics has two main advantages. First, when perceiving a scene, recognizing classes of objects is a difficult problem. Many objects that we classify together have very different appearances (e.g., “chair” or “table”). However, within a class, objects are designed to provide a specific set of functions, or potential interactions. Objects of the class “chair” offer a stable support for a human body, providing mainly a “sit-ability” affordance, even if two chairs may visually look very different. Objects of the class “cup” can hold liquids or small objects, providing a “contain-ability” affordance. These classes can gather several affordances (e.g., Objects of the class “table” provide “support-ability” for objects of smaller size, but also “sit-ability” for beings). Reasoning at the affordance level rather than at the visual features level allows for a more intuitive and consistent description of the environment. Second, such a high-level description is more easily interpretable, thus more easily communicable to other agents, but also more useful to the robot itself: by analyzing the environment, it directly knows which actions are possible, and where, without further processing, potentially simplifying the decision-making process.

In the past 20 years, this new approach to perception in robotics has gained attention, leading to an important amount of work and multiple surveys trying to give a structured understanding of the topic of Affordances in Robotics (Sahin et al., 2007; Zech et al., 2017; Jamone et al., 2018; Ardón et al., 2021).

Zech et al. (2017) give an extensive view of the modern research landscape on computational models of affordance in robotics. They point out that the field is both quite young and very active, and highlight the general trends that can be found in the published models: They usually adopt the agent's perspective as suggested by Sahin et al. (2007) and are evaluated on real robots; Exploration of the environment (e.g., interaction of the robot with an object) is the favored method to acquire affordance relations; These practices lead to good generalization capabilities of the models. Affordances are studied in a variety of tasks that covers well robot's required skills (manipulation, locomotion). On the other hand, models mostly use visual features as inputs, whereas other information about the environment would be useful (texture, weight, etc.). Offline learning is still present whereas online learning would allow more autonomous robots (but is difficult). Finally, models are aimed at robotic utility and rarely try to be biologically plausible.

In addition, Ardón et al. (2021) explicitly analyze the literature from the perspective of robot autonomy. They find that most works focus on supervised offline learning of probabilistic affordances with visual input features and primitive actions, quite consistently with Zech et al. (2017)'s findings. Their conclusion points to several research directions: Exploring more complex design choices (e.g., considering actions at the motion level); Using richer inputs, including context information as well as other physical properties of objects; Going toward more integrated affordance learning-and-using systems rather than systems that only detect affordances.

The papers published in this special issue contribute to the field on several fronts:

In “Automatic Generation of Object Shapes With Desired Affordances Using Voxelgrid Representation”, Andries et al. introduce an algorithm for generating object shapes with specified affordances. In particular, they use a variational autoencoder to learn a “function-to-form-mapping” for objects with particular affordances, where the encoder is responsible for transforming a 3D voxel grid representation of an object into latent variables that capture the shape-related functional properties of the input object which provides the affordances; the decoder then is responsible for transforming the latent variables into 3D voxel grid representations that can be used as a basis for detecting affordances in objects. The main goal of this article is to take a step toward automation of an object's design process. The use of affordances in this area shows significant potential.

In “Sensorimotor Contingencies as a Key Drive of Development: From Babies to Robots”, Jacquey et al. review the developmental psychology literature on sensorimotor contingency learning in human infants, and extract principles for cognitive robotics. For an autonomous agent, sensorimotor contingencies represent the link between its actions and the consequences of its actions. Typical psychology experiments involve babies wearing bracelets that produce sound when shaken, and investigate at which age human babies are able to discriminate which specific part of their bodies (e.g., left arm or right leg) shall be moved to produce sound, and which other parts shall not.

In human infants, sensorimotor contingency learning could facilitate the acquisition of reaching, develop manual exploration, and even lead them to explore task-specific (unusual) actions. More generally, this provides infants with progressively more accurate internal models of the actions' effects, permitting finer interactions with the surrounding world. Importantly, sensitivity to sensorimotor contingency appears to be one of the drives of development, contributing to the acquisition of four fundamental motor and cognitive abilities that the authors emphasize: body knowledge, memory, generalization and goal-directedness.

The authors then relate these principles to research on affordance learning and open-ended learning in robotics. They argue that sensitivity to sensorimotor contingencies is particularly important in developmental robotics because it provides a simple way to equip an agent with the ability to learn to interact with the world using self-organized exploration of its environment. They finally present a blueprint architecture demonstrating how exploitation of sensitivity to sensorimotor contingencies, combined with the notion of “goal”, could allow autonomous robots to further develop new sensorimotor skills. This architecture can serve as a guide for both the design of new computational models and the design of new empirical experiments aiming at testing model predictions.

In “Examples of Gibsonian affordances in legged robotics research using an empirical, generative framework”, Roberts et al. revisit through the use of Miracchi's *generative framework* how roboticists have already implicitly integrated affordances in the design of controllers. They analyse six recent works on legged locomotion in robotics to highlight how the controllers are designed to exploit the agent-environment interaction in order to take advantage of available affordances. In opposition to the tendency of having complex predictors for affordances, they promote the use of a combination of affordance-based reactive controllers with little to no internal representation, and focus the representational capabilities of the robot where the task requires it. This approach on affordances in robotics thus stand closer to Gibson's original definition.

In “Geometric affordance perception: leveraging deep 3D saliency with the Interaction Tensor”, Ruiz and Mayol-Cuevas present a real-time approach that predict multiple affordances simultaneously based on the geometry of the scene. For each desired type of affordance, they compute an Interaction Tensor between two objects based on a demonstration of the interaction. These single-affordance tensors are then merged and clustered into a multi-affordance descriptor, which is a pointcloud whose origin corresponds to one of the object of the demonstration scene. Detecting affordances in a new scene thus corresponds to re-align the pointcloud at each desired location. The authors then use the pointcloud to generate data in order to train an adapted version of the deep learning network PointNet++. They obtain in this way a predictor of affordances based on the scene saliency. Their approach balances the use of the geometrical properties of the scene with efficient learning.

In “Building an Affordances Map with Interactive Perception”, Le Goff et al. introduce an online method to learn affordance representation through autonomous exploration of the environment at the local level (object or environment parts) rather than the *meso* level (complete object) as most of the literature do. Their approach focus on building one relevance map per affordance as a probability of effect based on a supervoxel discretization of the environment. Each supervoxel describes an local area of the environment using a color histogram and a geometric histogram. They then train a classifier online to predict the occurrence or absence of the effect after applying an action on this area. Their algorithm selects one supervoxel to interact with based on the current relevance map, records the effects of the action (or their absence), then updates the relevance map, before selecting a new area to interact with. All the relevance maps are aggregated to produce the final affordance map and predict the affordances at the local level. Such online approaches are important in order to improve robot's autonomy.

## Conclusions

The concept of affordance has gained an increased interest by the robotics community in the last years. It provides (affords) roboticists with a theoretical framework that can both help design efficient solutions for robots' internal representations of the possible interactions with their environment, and help relate their models with the developmental psychology literature. The later can bring a vast source of inspiration from the way human infants progressively acquire more and more complex sensorimotor abilities and related internal representations of their surrounding world. It also helps draw bridges with the sensorimotor theory (O'Regan and Noë, 2001), which has contributed to a better understanding within the neuroscience community of how the human brain builds abstract internal representations from low-level sensorimotor regularities. We think that this line of research at the interface between cognitive robotics, developmental psychology and neuroscience paves the way toward more and more robust and efficient robot cognitive architectures, helping to progressively expand robots' cognitive (Thill et al., 2013; Renaudo et al., 2014; Santucci et al., 2016; Krichmar, 2018) and metacognitive (Verschure, 2016; Chatila et al., 2018) abilities.

## Author contributions

All authors listed have made a substantial, direct, and intellectual contribution to the work and approved it for publication.

## Funding

This research was funded in whole, or in part, by the French Agence Nationale de la Recherche (ANR) and the Austrian Science Fund (FWF) (ANR-21-CE33-0019-01/FWF: I 5755-N ELSA project) (MK and ER).

## Conflict of interest

The authors declare that the research was conducted in the absence of any commercial or financial relationships that could be construed as a potential conflict of interest.

## Publisher's note

All claims expressed in this article are solely those of the authors and do not necessarily represent those of their affiliated organizations, or those of the publisher, the editors and the reviewers. Any product that may be evaluated in this article, or claim that may be made by its manufacturer, is not guaranteed or endorsed by the publisher.

## References

[B1] Ardón P. Pairet É. Lohan K. Ramamoorthy S. Petrick R. (2021). Building affordance relations for robotic agents - a review, in Proceedings of the Thirtieth International Joint Conference on Artificial Intelligence (IJCAI-21) Survey Track, ed Zhou Z. H. (Montréal, QC), 4302–4311.

[B2] Arkin R. C. (1990). Integrating behavioral, perceptual, and world knowledge in reactive navigation. Robot. Auton. Syst. 6, 105–122. 10.1016/S0921-8890(05)80031-4

[B3] Brooks R. A. (1986). A robust layered control system for a mobile robot. IEEE J. Robot. Automat. 2, 14–23. 10.1109/JRA.1986.1087032

[B4] Chatila R. Renaudo E. Andries M. Chavez-Garcia R.-O. Luce-Vayrac P. Gottstein R.. (2018). Toward self-aware robots. Front. Robot. AI 5, 88. 10.3389/frobt.2018.00088 33500967 PMC7805649

[B5] Chemero A. (2003). An outline of a theory of affordances. Ecol. Psychol. 15, 181–195. 10.1207/S15326969ECO1502_5

[B6] Gibson J. J. (1979). The Ecological Approach to Visual Perception. Boston, MA: Houghton Mifflin.

[B7] Greeno J. G. (1994). Gibson's affordances. Psychol. Rev. 101, 336–342. 10.1037/0033-295X.101.2.336 8022965

[B8] Horton T. E. Chakraborty A. Amant R. S. (2012). Affordances for robots: a brief survey. AVANT. Pismo Awangardy Filozoficzno-Naukowej. 2, 70–84.

[B9] Jamone L. Ugur E. Cangelosi A. Fadiga L. Bernardino A. Piater J.. (2018). Affordances in psychology, neuroscience, and robotics: a survey. IEEE Trans. Cogn. Dev. Syst. 10, 4–25. 10.1109/TCDS.2016.2594134

[B10] Krichmar J. L. (2018). Neurorobotics–A thriving community and a promising pathway toward intelligent cognitive robots. Front. Neurorobot. 12, 42. 10.3389/fnbot.2018.00042 30061820 PMC6054919

[B11] Michaels C. F. (2003). Affordances: four points of debate. Ecol. Psychol. 15, 135–148. 10.1207/S15326969ECO1502_3

[B12] O'Regan J. K. Noë A. (2001). A sensorimotor account of vision and visual consciousness. Behav. Brain Sci. 24, 939–973. 10.1017/S0140525X01000115 12239892

[B13] Renaudo E. Girard B. Chatila R. Khamassi M. (2014). Design of a control architecture for habit learning in robots, in Conference on Biomimetic and Biohybrid Systems (Cham: Springer), 249–260. 10.1007/978-3-319-09435-9_22

[B14] Rizzolatti G. Fogassi L. Gallese V. (2001). Neurophysiological mechanisms underlying the understanding and imitation of action. Nat. Rev. Neurosci. 2, 661–670. 10.1038/35090060 11533734

[B15] Sahin E. Cakmak M. Dogar M. Ugur E. Ucoluk G. (2007). To afford or not to afford: a new formalization of affordances toward affordance-based robot control. Adapt. Behav. 15, 447–472. 10.1177/1059712307084689

[B16] Sanders J. T. (1997). An ontology of affordances. Ecol. Psychol. 9, 97–112. 10.1207/s15326969eco0901_4

[B17] Santucci V. G. Baldassarre G. Mirolli M. (2016). Grail: a goal-discovering robotic architecture for intrinsically-motivated learning. IEEE Trans. Cogn. Dev. Syst. 8, 214–231. 10.1109/TCDS.2016.2538961

[B18] Steedman M. (2002). Formalizing affordance, in Proceedings of the Annual Meeting of the Cognitive Science Society (New York, NY).

[B19] Stoffregen T. A. (2003). Affordances as properties of the animal-environment system. Ecol. Psychol. 15, 115–134. 10.1207/S15326969ECO1502_2

[B20] Thill S. Caligiore D. Borghi A. M. Ziemke T. Baldassarre G. (2013). Theories and computational models of affordance and mirror systems: an integrative review. Neurosci. Biobehav. Rev. 37, 491–521. 10.1016/j.neubiorev.2013.01.012 23333761

[B21] Turvey M. T. (1992). Affordances and prospective control: an outline of the ontology. Ecol. Psychol. 4(3):173–187. 10.1207/s15326969eco0403_3

[B22] Verschure P. F. (2016). Synthetic consciousness: the distributed adaptive control perspective. Philos. Trans. R. Soc. B Biol. Sci. 371, 20150448. 10.1098/rstb.2015.0448 27431526 PMC4958942

[B23] Wells A. J. (2002). Gibson's affordances and turing's theory of computation. Ecol. Psychol. 14, 140–180. 10.1207/S15326969ECO1403_3

[B24] Zech P. Haller S. Rezapour Lakani S. Ridge B. Ugur E. Piater J. (2017). Computational models of affordance in robotics: a taxonomy and systematic classification. Adapt. Behav. 25, 235–271. 10.1177/1059712317726357

